# Low-temperature phase of hexaguanidinium hepta­molybdate monohydrate

**DOI:** 10.1107/S1600536808008234

**Published:** 2008-04-02

**Authors:** Santiago Reinoso, Michael H. Dickman, Antonia Praetorius, Ulrich Kortz*

**Affiliations:** a School of Engineering and Science, Jacobs University Bremen, PO Box 750561, 28725 Bremen, Germany

## Abstract

The crystal structure of the title compound, [C(NH_2_)_3_]_6_[Mo_7_O_24_]·H_2_O, previously determined at room temperature in the monoclinic space group *C*2/*c* from Weissenberg techniques [Don & Weakley (1981 ▶). *Acta Cryst*. B**37**, 451–453], has been redetermined from low-temperature single-crystal data in the monoclinic space group *P*2_1_/*c*. The asymmetric unit contains one hepta­molybdate anion, six guanidinium cations and one water mol­ecule of hydration. The anions and cations are linked by an extensive network of N—H⋯O hydrogen bonds.

## Related literature

For the previous determination of the title compound in the monoclinic space group *C*2/*c*, see: Don & Weakley (1981 ▶). For an example of a structurally characterized [Mo_7_O_24_]^6−^ anion, see: Kortz & Pope (1995 ▶). For more information about isopolymolybdates and polyoxometalates in general, see: Pope (1983 ▶).
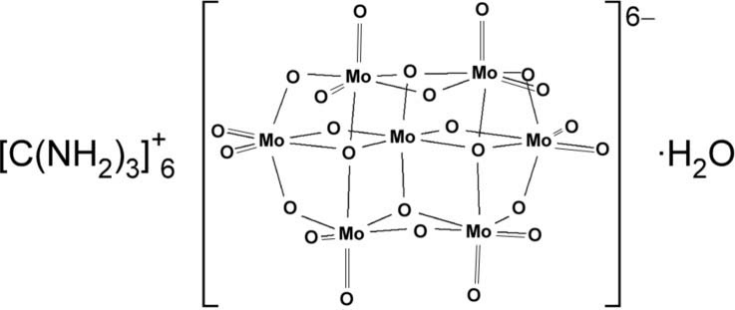


## Experimental

### 

#### Crystal data

(CH_6_N_3_)_6_[Mo_7_O_24_].H_2_O
*M* *_r_* = 1434.12
Monoclinic, 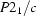

*a* = 11.9402 (6) Å
*b* = 15.9131 (9) Å
*c* = 19.8223 (13) Å
β = 92.312 (4)°
*V* = 3763.3 (4) Å^3^
*Z* = 4
Mo *K*α radiation
μ = 2.37 mm^−1^
*T* = 173 (2) K
0.17 × 0.17 × 0.08 mm

#### Data collection

Bruker X8 APEXII CCD area-detector diffractometer
Absorption correction: multi-scan (*APEX2*; Bruker, 2005 ▶) *T*_min_ = 0.689, *T*_max_ = 0.833
153838 measured reflections
15842 independent reflections
9689 reflections with *I* > 2s(*I*)
*R*_int_ = 0.150

#### Refinement

*R*[*F* ^2^ > 2σ(*F* ^2^)] = 0.053
*wR*(*F* ^2^) = 0.164
*S* = 1.05
15842 reflections
506 parameters
H-atom parameters constrained
Δρ_max_ = 1.77 e Å^−3^
Δρ_min_ = −2.33 e Å^−3^

### 

Data collection: *APEX2* (Bruker, 2005 ▶); cell refinement: *APEX2*; data reduction: *APEX2*; program(s) used to solve structure: *SHELXTL* (Sheldrick, 2008 ▶); program(s) used to refine structure: *SHELXTL*; molecular graphics: *SHELXTL*; software used to prepare material for publication: *SHELXTL* and *WINGX* Farrugia (1999 ▶)..

## Supplementary Material

Crystal structure: contains datablocks global, I. DOI: 10.1107/S1600536808008234/mg2049sup1.cif

Structure factors: contains datablocks I. DOI: 10.1107/S1600536808008234/mg2049Isup2.hkl

Additional supplementary materials:  crystallographic information; 3D view; checkCIF report

## Figures and Tables

**Table 1:** Hydrogen-bond geometry (Å, °)

*D*—H⋯*A*	*D*—H	H⋯*A*	*D*⋯*A*	*D*—H⋯*A*
N11—H11*A*⋯O1*W*	0.88	1.90	2.765 (6)	168
N11—H11*B*⋯O3*A*	0.88	2.35	3.091 (6)	142
N12—H12*A*⋯O2*A*^i^	0.88	2.00	2.845 (5)	160
N12—H12*B*⋯O5*B*	0.88	2.14	2.903 (5)	145
N13—H13*A*⋯O6*A*^ii^	0.88	1.96	2.828 (5)	169
N13—H13*B*⋯O3*B*	0.88	2.23	2.930 (5)	136
N14—H14*B*⋯O13	0.88	1.93	2.774 (4)	159
N14—H14*A*⋯O57^iii^	0.88	1.98	2.814 (4)	159
N15—H15*A*⋯O2*B*^iv^	0.88	2.03	2.843 (5)	153
N15—H15*B*⋯O3*A*^v^	0.88	2.13	2.866 (5)	141
N15—H15*B*⋯O34^v^	0.88	2.62	3.302 (5)	135
N16—H16*B*⋯O1*A*^ii^	0.88	2.13	2.967 (5)	159
N16—H16*A*⋯O45^vi^	0.88	2.18	2.977 (5)	151
N21—H21*A*⋯O1*B*^vi^	0.88	2.18	3.006 (5)	156
N21—H21*A*⋯O2*B*^vi^	0.88	2.40	2.926 (5)	118
N21—H21*B*⋯O3*A*	0.88	2.22	2.992 (5)	147
N22—H22*A*⋯O36^vii^	0.88	2.32	3.087 (5)	145
N22—H22*A*⋯O467^vii^	0.88	2.50	3.216 (5)	139
N22—H22*A*⋯O6*B*^vii^	0.88	2.64	3.290 (5)	131
N22—H22*B*⋯O25	0.88	2.10	2.977 (5)	177
N23—H23*A*⋯O36	0.88	2.07	2.937 (5)	169
N23—H23*B*⋯O25^vi^	0.88	2.26	3.023 (5)	145
N23—H23*B*⋯O124^vi^	0.88	2.50	3.219 (5)	140
N24—H24*A*⋯O13	0.88	2.45	3.154 (5)	137
N24—H24*A*⋯O3*A*	0.88	2.50	3.186 (5)	135
N24—H24*B*⋯O12^viii^	0.88	2.09	2.855 (5)	145
N25—H25*A*⋯O34^v^	0.88	2.22	2.990 (5)	146
N25—H25*B*⋯O7*A*^iii^	0.88	2.08	2.931 (5)	162
N26—H26*A*⋯O3*B*^ii^	0.88	1.98	2.859 (5)	175
N26—H26*B*⋯O1*W*^ii^	0.88	2.50	3.084 (6)	124
N26—H26*B*⋯O6*A*	0.88	2.57	3.197 (5)	129
N31—H31*A*⋯O1*B*^vi^	0.88	2.50	3.248 (6)	143
N31—H31*B*⋯O5*A*^ix^	0.88	2.38	3.188 (6)	152
N32—H32*A*⋯N34^x^	0.88	2.50	3.242 (6)	143
N32—H32*B*⋯O467^vii^	0.88	2.02	2.864 (5)	160
N33—H33*A*⋯N24^vi^	0.88	2.60	3.334 (6)	142
N33—H33*B*⋯O124^vi^	0.88	2.02	2.867 (5)	160
N34—H34*B*⋯O67^viii^	0.88	1.94	2.776 (5)	157
N34—H34*A*⋯O57^iii^	0.88	2.44	3.154 (5)	139
N34—H34*A*⋯O5*A*^iii^	0.88	2.55	3.195 (5)	131
N35—H35*A*⋯O2*A*^iv^	0.88	2.29	3.040 (5)	144
N35—H35*B*⋯O5*B*^iii^	0.88	1.98	2.849 (5)	170
N36—H36*A*⋯O5*A*^vi^	0.88	2.16	2.913 (5)	144
N36—H36*B*⋯O6*B*	0.88	2.13	2.868 (5)	141
O1*W*—H1*OW*⋯O6*B*^iii^	0.86	2.22	2.948 (5)	142
O1*W*—H1*OW*⋯O7*B*^iii^	0.86	2.44	3.086 (5)	133
O1*W*—H2*OW*⋯O5*A*^ix^	0.86	2.35	2.973 (5)	130

Symmetry codes: (i) 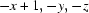; (ii) 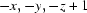; (iii) 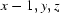; (iv) 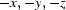; (v) 
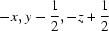
; (vi) 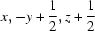; (vii) 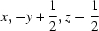; (viii) 
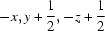
; (ix) 
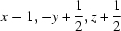
; (x) 
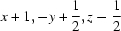
.

**Fig. 1.**
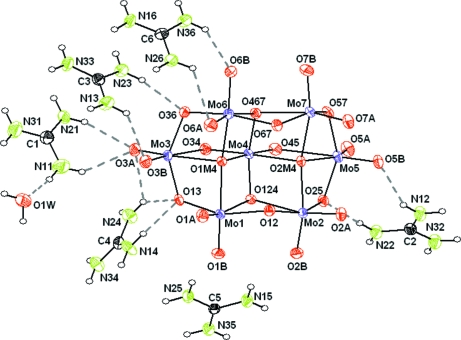
ORTEP plot of (I) showing 50% probability displacement ellipsoids.

## supplementary crystallographic information

### Comment

Acidification of aqueous molybdate solutions gives rise to the formation of different isopolymolybdate(VI) anions depending on the pH. The heptamolybdate anion, [Mo_7_O_24_]^6-^, is known to be the predominant species in neutral to moderately acidic solutions (Pope, 1983). Single crystals of the title compound, [C(NH_2_)_3_]_6_[Mo_7_O_24_].H_2_O (I), were obtained in an attempt to prepare a guanidinium salt of a dimethyltin-containing phosphomolybdate species.

Routine examination of (I) at low temperature resulted in the assignment of the monoclinic space group *P*2_1_/*c*, with the C-centered reflections being systematically weak. However, a previous room-temperature study using Weissenberg techniques reported the structure of (I) in the monoclinic space group *C*2/*c* (Don & Weakley, 1981). Therefore, data were collected on a second crystal at room temperature, confirming the original assignment of *C*2/*c*; data were then collected on this same crystal cooled to 173 K, confirming the low-temperature assignment of *P*2_1_/*c*. The value of *R*_int_ for the low-temperature data was 15.0%. We attribute this higher than usual value to the phase change; the value of *R*_int_ for the room-temperature data was less than 10%. The low-temperature structure consists of one [Mo_7_O_24_]^6-^ anion, six [C(NH_2_)_3_]^+^ cations, and one molecule of water of hydration in the asymmetric unit (Fig. 1). The [Mo_7_O_24_]^6-^ anion shows the well known bent arrangement of seven edge-sharing MoO_6_ distorted octahedra (Kortz & Pope, 1995), which can be formally derived from the parent {*M*_10_O_28_} decametalate framework by removal of three octahedra from the central level (Pope, 1983). The anions and the cations are linked by an extensive and intricate three-dimensional network of N—H···O hydrogen bonds involving all guanidinium –NH_2_ groups and all heptamolybdate terminal and bridging O atoms, with the exception of the terminal O7B and the bridging µ_4_-O atoms (O1M4 and O2M4).

### Experimental

To an aqueous solution (30 ml) of Na_2_MoO_4_.2H_2_O (0.726 g) and Na_2_HPO_4_ (0.071 g) was added solid (CH_3_)SnCl_2_ (0.220 g). After stirring for 30 min at room temperature, few drops of aqueous 1*M* [C(NH_2_)_3_]Cl were added, and the resulting solution was left to slowly evaporate at room temperature, colorless block-like single crystals of the title compound forming in a few days.

### Refinement

The H atoms of the guanidinium cations were positioned geometrically and refined as riding. The positional parameters of the H atoms of the water molecule were calculated using the *CALC*OH tool of *WinGX* (Farrugia, 1999) and fixed during the refinment. All H atoms were refined with isotropic displacement parameters fixed at 1.2 times *U*_eq_ of their parent atoms.

### Crystal data

**Table:**

(C_1_H_6_N_3_)_6_[Mo_7_O_24_]·H_2_O	*F*_000_ = 2776
*M**_r_* = 1434.12	*D*_x_ = 2.531 Mg m^−^^3^
Monoclinic, *P*2_1_/*c*	Mo *K*α radiation λ = 0.71073 Å
Hall symbol: -P 2ybc	Cell parameters from 7762 reflections
*a* = 11.9402 (6) Å	θ = 2.3–29.7º
*b* = 15.9131 (9) Å	µ = 2.37 mm^−^^1^
*c* = 19.8223 (13) Å	*T* = 173 (2) K
β = 92.312 (4)º	Prism, colourless
*V* = 3763.3 (4) Å^3^	0.17 × 0.17 × 0.08 mm
*Z* = 4	

### Data collection

**Table:**

Bruker X8 APEXII CCD area-detector diffractometer	15842 independent reflections
Radiation source: sealed tube	9689 reflections with *I* > 2s(*I*)
Monochromator: graphite	*R*_int_ = 0.150
*T* = 173(2) K	θ_max_ = 34.4º
φ and ω scans	θ_min_ = 1.7º
Absorption correction: multi-scanAPEXII (Bruker, 2005)	*h* = −18→18
*T*_min_ = 0.689, *T*_max_ = 0.833	*k* = −25→25
153838 measured reflections	*l* = −31→31

### Refinement

**Table:**

Refinement on *F*^2^	H-atom parameters constrained
Least-squares matrix: full	*w* = 1/[σ^2^(*F*_o_^2^) + (0.065*P*)^2^ + 1.0536*P*] where *P* = (*F*_o_^2^ + 2*F*_c_^2^)/3
*R*[*F*^2^ > 2σ(*F*^2^)] = 0.053	(Δ/σ)_max_ = 0.001
*wR*(*F*^2^) = 0.164	Δρ_max_ = 1.77 e Å^−^^3^
*S* = 1.06	Δρ_min_ = −2.32 e Å^−^^3^
15842 reflections	Extinction correction: SHELXL
506 parameters	Extinction coefficient: 0.00102 (9)

### Special details

**Table:**

Geometry. All e.s.d.'s (except the e.s.d. in the dihedral angle between two l.s. planes) are estimated using the full covariance matrix. The cell e.s.d.'s are taken into account individually in the estimation of e.s.d.'s in distances, angles and torsion angles; correlations between e.s.d.'s in cell parameters are only used when they are defined by crystal symmetry. An approximate (isotropic) treatment of cell e.s.d.'s is used for estimating e.s.d.'s involving l.s. planes.
Refinement. Refinement of *F*^2^ against ALL reflections. The weighted *R*-factor *wR* and goodness of fit *S* are based on *F*^2^, conventional *R*-factors *R* are based on *F*, with *F* set to zero for negative *F*^2^. The threshold expression of *F*^2^ > σ(*F*^2^) is used only for calculating *R*-factors(gt) *etc*. and is not relevant to the choice of reflections for refinement. *R*-factors based on *F*^2^ are statistically about twice as large as those based on *F*, and *R*- factors based on ALL data will be even larger.

### Fractional atomic coordinates and isotropic or equivalent isotropic displacement parameters (Å^2^)

**Table:**

	*x*	*y*	*z*	*U*_iso_*/*U*_eq_	
Mo1	0.02068 (3)	0.06939 (2)	0.221800 (17)	0.01551 (8)	
Mo2	0.22006 (3)	0.06307 (2)	0.115322 (17)	0.01597 (8)	
Mo3	0.04258 (3)	0.17323 (2)	0.359758 (18)	0.01829 (9)	
Mo4	0.24555 (3)	0.20215 (2)	0.245700 (16)	0.01436 (8)	
Mo5	0.44752 (3)	0.16970 (2)	0.131721 (18)	0.01815 (9)	
Mo6	0.26730 (3)	0.06331 (2)	0.377162 (17)	0.01662 (8)	
Mo7	0.46759 (3)	0.06731 (2)	0.270582 (18)	0.01634 (8)	
O1B	−0.0700 (3)	0.0900 (2)	0.15428 (15)	0.0235 (7)	
O1A	−0.0382 (3)	−0.0105 (2)	0.26587 (16)	0.0249 (7)	
O2B	0.1171 (3)	0.0798 (2)	0.05310 (15)	0.0238 (7)	
O2A	0.2968 (3)	−0.0190 (2)	0.08550 (16)	0.0246 (7)	
O3B	−0.0348 (3)	0.1000 (2)	0.40054 (16)	0.0270 (7)	
O3A	−0.0090 (3)	0.2697 (2)	0.38553 (16)	0.0266 (7)	
O5B	0.5214 (3)	0.0936 (2)	0.09095 (16)	0.0256 (7)	
O5A	0.5025 (3)	0.2644 (2)	0.10420 (17)	0.0271 (7)	
O6B	0.3707 (3)	0.0785 (2)	0.43900 (15)	0.0241 (7)	
O6A	0.1877 (3)	−0.0173 (2)	0.40575 (16)	0.0269 (7)	
O7B	0.5567 (3)	0.0861 (2)	0.33821 (16)	0.0248 (7)	
O7A	0.5255 (3)	−0.0139 (2)	0.22726 (17)	0.0256 (7)	
O12	0.1370 (2)	0.00412 (18)	0.18077 (14)	0.0186 (6)	
O13	−0.0327 (2)	0.16739 (19)	0.27278 (15)	0.0201 (6)	
O1M4	0.1493 (2)	0.10064 (18)	0.29826 (13)	0.0156 (5)	
O2M4	0.3391 (2)	0.09888 (18)	0.19337 (13)	0.0152 (5)	
O25	0.3103 (2)	0.16081 (19)	0.08014 (14)	0.0203 (6)	
O34	0.1626 (3)	0.26978 (19)	0.29144 (15)	0.0215 (6)	
O36	0.1794 (2)	0.16226 (19)	0.41150 (14)	0.0208 (6)	
O45	0.3298 (3)	0.26854 (19)	0.19892 (15)	0.0216 (6)	
O57	0.5234 (2)	0.16324 (19)	0.21861 (15)	0.0205 (6)	
O67	0.3490 (2)	0.00393 (18)	0.31125 (14)	0.0184 (6)	
O124	0.1415 (2)	0.16496 (18)	0.17796 (14)	0.0160 (5)	
O467	0.3486 (2)	0.16389 (18)	0.31372 (14)	0.0175 (6)	
C1	−0.1887 (4)	0.2916 (3)	0.5167 (3)	0.0331 (11)	
C2	0.4571 (4)	0.1742 (3)	−0.0860 (2)	0.0231 (9)	
C3	0.0480 (4)	0.1669 (3)	0.5786 (2)	0.0227 (9)	
C4	−0.2559 (3)	0.2966 (3)	0.24617 (19)	0.0216 (9)	
C5	−0.2245 (4)	−0.0488 (3)	0.1085 (2)	0.0199 (8)	
C6	0.2837 (4)	0.0415 (3)	0.6044 (2)	0.0225 (9)	
N11	−0.2273 (4)	0.2477 (3)	0.4624 (3)	0.0513 (14)	
H11A	−0.2897	0.2186	0.4643	0.062*	
H11B	−0.1900	0.2481	0.4250	0.062*	
N12	0.5055 (3)	0.1087 (3)	−0.0551 (2)	0.0288 (9)	
H12A	0.5597	0.0814	−0.0744	0.035*	
H12B	0.4833	0.0927	−0.0153	0.035*	
N13	0.0031 (4)	0.1011 (3)	0.5475 (2)	0.0308 (9)	
H13A	−0.0506	0.0726	0.5663	0.037*	
H13B	0.0267	0.0857	0.5079	0.037*	
N14	−0.2542 (3)	0.2142 (3)	0.24754 (19)	0.0298 (10)	
H14A	−0.3154	0.1856	0.2369	0.036*	
H14B	−0.1917	0.1875	0.2591	0.036*	
N15	−0.1334 (3)	−0.0878 (3)	0.0894 (2)	0.0275 (9)	
H15A	−0.1071	−0.0776	0.0494	0.033*	
H15B	−0.0990	−0.1238	0.1168	0.033*	
N16	0.2399 (3)	0.0624 (3)	0.66201 (18)	0.0269 (9)	
H16A	0.2705	0.1028	0.6869	0.032*	
H16B	0.1801	0.0361	0.6756	0.032*	
N21	−0.0947 (3)	0.3375 (3)	0.5144 (2)	0.0349 (11)	
H21A	−0.0712	0.3667	0.5500	0.042*	
H21B	−0.0566	0.3385	0.4773	0.042*	
N22	0.3746 (3)	0.2148 (2)	−0.05681 (19)	0.0274 (9)	
H22A	0.3420	0.2579	−0.0773	0.033*	
H22B	0.3526	0.1986	−0.0170	0.033*	
N23	0.1296 (3)	0.2099 (2)	0.55026 (19)	0.0253 (8)	
H23A	0.1536	0.1944	0.5108	0.030*	
H23B	0.1596	0.2538	0.5711	0.030*	
N24	−0.1629 (3)	0.3397 (2)	0.2631 (2)	0.0299 (9)	
H24A	−0.1009	0.3128	0.2751	0.036*	
H24B	−0.1638	0.3950	0.2622	0.036*	
N25	−0.2628 (3)	−0.0643 (3)	0.16922 (19)	0.0266 (8)	
H25A	−0.2275	−0.1002	0.1963	0.032*	
H25B	−0.3234	−0.0386	0.1824	0.032*	
N26	0.2384 (3)	−0.0186 (2)	0.5654 (2)	0.0273 (8)	
H26A	0.1780	−0.0451	0.5779	0.033*	
H26B	0.2687	−0.0317	0.5270	0.033*	
N31	−0.2477 (4)	0.2892 (3)	0.5725 (2)	0.0454 (12)	
H31A	−0.2252	0.3180	0.6084	0.054*	
H31B	−0.3092	0.2588	0.5734	0.054*	
N32	0.4905 (4)	0.1984 (3)	−0.1459 (2)	0.0301 (9)	
H32A	0.5447	0.1712	−0.1652	0.036*	
H32B	0.4584	0.2418	−0.1663	0.036*	
N33	0.0121 (4)	0.1901 (3)	0.63845 (19)	0.0292 (9)	
H33A	−0.0416	0.1616	0.6572	0.035*	
H33B	0.0420	0.2340	0.6593	0.035*	
N34	−0.3495 (3)	0.3368 (2)	0.2289 (2)	0.0281 (9)	
H34A	−0.4108	0.3083	0.2183	0.034*	
H34B	−0.3504	0.3921	0.2280	0.034*	
N35	−0.2762 (3)	0.0054 (2)	0.06749 (19)	0.0268 (8)	
H35A	−0.2500	0.0155	0.0274	0.032*	
H35B	−0.3369	0.0313	0.0802	0.032*	
N36	0.3742 (3)	0.0814 (3)	0.58373 (19)	0.0269 (9)	
H36A	0.4049	0.1217	0.6086	0.032*	
H36B	0.4034	0.0673	0.5452	0.032*	
O1W	−0.4098 (3)	0.1463 (3)	0.4856 (2)	0.0446 (10)	
H1OW	−0.4589	0.1330	0.4545	0.054*	
H2OW	−0.4419	0.1380	0.5233	0.054*	

### Atomic displacement parameters (Å^2^)

**Table:**

	*U*^11^	*U*^22^	*U*^33^	*U*^12^	*U*^13^	*U*^23^
Mo1	0.01223 (16)	0.01668 (17)	0.01772 (15)	−0.00120 (12)	0.00174 (12)	−0.00124 (12)
Mo2	0.01468 (17)	0.01756 (17)	0.01579 (15)	0.00145 (13)	0.00233 (12)	−0.00065 (12)
Mo3	0.01455 (17)	0.02142 (19)	0.01925 (16)	−0.00144 (13)	0.00494 (13)	−0.00450 (13)
Mo4	0.01179 (16)	0.01357 (16)	0.01786 (15)	0.00013 (12)	0.00221 (12)	0.00014 (12)
Mo5	0.01378 (17)	0.01978 (19)	0.02126 (17)	0.00092 (13)	0.00547 (13)	0.00439 (13)
Mo6	0.01591 (17)	0.01855 (18)	0.01550 (15)	−0.00138 (13)	0.00215 (12)	0.00095 (12)
Mo7	0.01257 (16)	0.01696 (17)	0.01955 (16)	0.00162 (12)	0.00167 (12)	0.00165 (12)
O1B	0.0211 (16)	0.0266 (17)	0.0224 (14)	0.0034 (13)	−0.0041 (12)	0.0003 (12)
O1A	0.0231 (16)	0.0247 (17)	0.0272 (16)	−0.0041 (13)	0.0043 (13)	0.0016 (13)
O2B	0.0231 (16)	0.0286 (17)	0.0196 (14)	−0.0017 (13)	−0.0012 (12)	0.0002 (12)
O2A	0.0238 (16)	0.0236 (16)	0.0269 (16)	0.0061 (13)	0.0066 (13)	−0.0020 (13)
O3B	0.0231 (17)	0.0334 (19)	0.0250 (15)	−0.0062 (14)	0.0072 (13)	−0.0005 (14)
O3A	0.0227 (16)	0.0247 (17)	0.0326 (17)	−0.0006 (13)	0.0054 (13)	−0.0126 (13)
O5B	0.0198 (16)	0.0301 (18)	0.0274 (16)	0.0055 (13)	0.0089 (13)	0.0005 (13)
O5A	0.0251 (17)	0.0239 (17)	0.0328 (17)	−0.0002 (14)	0.0080 (14)	0.0125 (13)
O6B	0.0224 (16)	0.0309 (18)	0.0191 (14)	0.0006 (13)	0.0009 (12)	−0.0004 (12)
O6A	0.0265 (17)	0.0263 (17)	0.0281 (16)	−0.0050 (14)	0.0054 (14)	0.0031 (13)
O7B	0.0205 (16)	0.0263 (17)	0.0274 (16)	0.0008 (13)	−0.0036 (13)	0.0049 (13)
O7A	0.0224 (16)	0.0227 (16)	0.0323 (17)	0.0052 (13)	0.0074 (13)	0.0033 (13)
O12	0.0198 (15)	0.0160 (14)	0.0201 (13)	0.0007 (11)	0.0021 (11)	−0.0017 (11)
O13	0.0118 (13)	0.0249 (16)	0.0239 (14)	0.0013 (12)	0.0040 (11)	−0.0038 (12)
O1M4	0.0137 (14)	0.0170 (14)	0.0163 (12)	−0.0003 (11)	0.0018 (10)	−0.0002 (10)
O2M4	0.0135 (13)	0.0157 (13)	0.0169 (12)	0.0014 (11)	0.0048 (10)	0.0018 (10)
O25	0.0175 (15)	0.0258 (16)	0.0179 (13)	0.0009 (12)	0.0045 (11)	0.0038 (11)
O34	0.0189 (15)	0.0204 (15)	0.0254 (15)	0.0003 (12)	0.0025 (12)	−0.0039 (12)
O36	0.0191 (15)	0.0235 (16)	0.0201 (14)	−0.0012 (12)	0.0029 (11)	−0.0057 (11)
O45	0.0215 (15)	0.0178 (15)	0.0255 (15)	−0.0038 (12)	0.0019 (12)	0.0044 (12)
O57	0.0118 (13)	0.0215 (16)	0.0283 (15)	−0.0008 (11)	0.0027 (12)	0.0050 (12)
O67	0.0171 (14)	0.0159 (14)	0.0225 (14)	−0.0009 (11)	0.0041 (11)	0.0016 (11)
O124	0.0144 (13)	0.0164 (14)	0.0174 (12)	−0.0009 (11)	0.0006 (10)	0.0004 (10)
O467	0.0151 (14)	0.0194 (15)	0.0179 (13)	−0.0005 (11)	0.0017 (11)	0.0017 (11)
C1	0.030 (3)	0.037 (3)	0.032 (2)	0.005 (2)	−0.001 (2)	−0.007 (2)
C2	0.021 (2)	0.022 (2)	0.027 (2)	0.0038 (17)	−0.0003 (17)	−0.0043 (17)
C3	0.021 (2)	0.021 (2)	0.026 (2)	0.0001 (17)	0.0010 (17)	0.0003 (16)
C4	0.023 (2)	0.020 (2)	0.0227 (19)	−0.0002 (17)	0.0064 (17)	−0.0016 (16)
C5	0.020 (2)	0.019 (2)	0.0199 (18)	0.0045 (16)	−0.0018 (16)	−0.0040 (15)
C6	0.027 (2)	0.020 (2)	0.0203 (18)	−0.0015 (18)	−0.0011 (17)	0.0021 (16)
N11	0.044 (3)	0.066 (4)	0.044 (3)	−0.018 (3)	0.004 (2)	−0.017 (3)
N12	0.029 (2)	0.029 (2)	0.0289 (19)	0.0158 (17)	0.0043 (17)	0.0043 (16)
N13	0.037 (2)	0.025 (2)	0.031 (2)	−0.0157 (18)	0.0049 (18)	−0.0070 (16)
N14	0.0161 (19)	0.0166 (19)	0.056 (3)	−0.0017 (14)	−0.0017 (19)	0.0027 (17)
N15	0.027 (2)	0.030 (2)	0.0255 (18)	0.0131 (17)	0.0029 (16)	−0.0001 (16)
N16	0.029 (2)	0.032 (2)	0.0203 (17)	−0.0072 (17)	0.0101 (15)	−0.0042 (15)
N21	0.0174 (19)	0.050 (3)	0.038 (2)	−0.0126 (19)	0.0076 (17)	−0.016 (2)
N22	0.029 (2)	0.027 (2)	0.0259 (18)	0.0129 (17)	0.0019 (16)	0.0031 (15)
N23	0.028 (2)	0.0222 (19)	0.0256 (18)	−0.0116 (16)	0.0055 (16)	−0.0008 (15)
N24	0.025 (2)	0.0193 (19)	0.045 (2)	−0.0076 (16)	0.0025 (18)	−0.0027 (17)
N25	0.023 (2)	0.034 (2)	0.0229 (18)	0.0077 (17)	0.0041 (15)	0.0004 (15)
N26	0.0222 (19)	0.032 (2)	0.0276 (19)	−0.0117 (17)	0.0050 (15)	−0.0039 (16)
N31	0.043 (3)	0.059 (3)	0.036 (2)	−0.009 (2)	0.014 (2)	−0.011 (2)
N32	0.031 (2)	0.032 (2)	0.0278 (19)	0.0099 (18)	0.0049 (17)	0.0057 (17)
N33	0.031 (2)	0.033 (2)	0.0240 (18)	−0.0061 (18)	0.0097 (16)	−0.0054 (16)
N34	0.025 (2)	0.0180 (19)	0.042 (2)	0.0055 (15)	0.0019 (17)	0.0034 (16)
N35	0.025 (2)	0.030 (2)	0.0249 (18)	0.0118 (17)	0.0001 (15)	−0.0004 (16)
N36	0.030 (2)	0.028 (2)	0.0230 (18)	−0.0130 (17)	0.0045 (16)	−0.0012 (15)
O1W	0.027 (2)	0.058 (3)	0.049 (2)	−0.0094 (19)	0.0001 (17)	−0.016 (2)

### Geometric parameters (Å, °)

**Table:**

Mo1—O1A	1.710 (3)	C3—N13	1.317 (6)
Mo1—O1B	1.719 (3)	C3—N33	1.330 (6)
Mo1—O12	1.939 (3)	C3—N23	1.334 (6)
Mo1—O13	1.978 (3)	C4—N14	1.311 (6)
Mo1—O1M4	2.171 (3)	C4—N34	1.321 (6)
Mo1—O124	2.291 (3)	C4—N24	1.336 (6)
Mo1—Mo3	3.1968 (5)	C5—N15	1.320 (5)
Mo2—O2A	1.714 (3)	C5—N35	1.321 (5)
Mo2—O2B	1.726 (3)	C5—N25	1.328 (5)
Mo2—O12	1.910 (3)	C6—N16	1.318 (5)
Mo2—O25	2.032 (3)	C6—N36	1.332 (6)
Mo2—O2M4	2.136 (3)	C6—N26	1.331 (6)
Mo2—O124	2.268 (3)	N11—H11A	0.8800
Mo2—Mo5	3.2078 (5)	N11—H11B	0.8800
Mo3—O3B	1.711 (3)	N12—H12A	0.8800
Mo3—O3A	1.738 (3)	N12—H12B	0.8800
Mo3—O36	1.902 (3)	N13—H13A	0.8800
Mo3—O13	1.914 (3)	N13—H13B	0.8800
Mo3—O1M4	2.138 (3)	N14—H14A	0.8800
Mo3—Mo6	3.2099 (5)	N14—H14B	0.8800
Mo4—O34	1.742 (3)	N15—H15A	0.8800
Mo4—O45	1.751 (3)	N15—H15B	0.8800
Mo4—O124	1.887 (3)	N16—H16A	0.8800
Mo4—O467	1.889 (3)	N16—H16B	0.8800
Mo4—O1M4	2.261 (3)	N21—H21A	0.8800
Mo4—O2M4	2.262 (3)	N21—H21B	0.8800
Mo5—O5B	1.719 (3)	N22—H22A	0.8800
Mo5—O5A	1.740 (3)	N22—H22B	0.8800
Mo5—O25	1.901 (3)	N23—H23A	0.8800
Mo5—O57	1.916 (3)	N23—H23B	0.8800
Mo5—O2M4	2.137 (3)	N24—H24A	0.8800
Mo5—Mo7	3.1993 (5)	N24—H24B	0.8800
Mo6—O6A	1.708 (3)	N25—H25A	0.8800
Mo6—O6B	1.721 (3)	N25—H25B	0.8800
Mo6—O67	1.911 (3)	N26—H26A	0.8800
Mo6—O36	2.025 (3)	N26—H26B	0.8800
Mo6—O1M4	2.146 (3)	N31—H31A	0.8800
Mo6—O467	2.277 (3)	N31—H31B	0.8800
Mo7—O7B	1.704 (3)	N32—H32A	0.8800
Mo7—O7A	1.713 (3)	N32—H32B	0.8800
Mo7—O67	1.941 (3)	N33—H33A	0.8800
Mo7—O57	1.973 (3)	N33—H33B	0.8800
Mo7—O2M4	2.181 (3)	N34—H34A	0.8800
Mo7—O467	2.282 (3)	N34—H34B	0.8800
C1—N31	1.336 (6)	N35—H35A	0.8800
C1—N21	1.342 (6)	N35—H35B	0.8800
C1—N11	1.350 (7)	N36—H36A	0.8800
C2—N32	1.326 (6)	N36—H36B	0.8800
C2—N12	1.328 (5)	O1W—H1OW	0.8600
C2—N22	1.330 (6)	O1W—H2OW	0.8620
			
?···?	?		
			
O1A—Mo1—O1B	106.28 (16)	O467—Mo6—Mo3	86.03 (7)
O1A—Mo1—O12	97.62 (14)	O7B—Mo7—O7A	105.95 (16)
O1B—Mo1—O12	102.41 (14)	O7B—Mo7—O67	101.99 (14)
O1A—Mo1—O13	100.22 (14)	O7A—Mo7—O67	97.60 (14)
O1B—Mo1—O13	92.39 (14)	O7B—Mo7—O57	93.52 (14)
O12—Mo1—O13	152.57 (12)	O7A—Mo7—O57	99.81 (14)
O1A—Mo1—O1M4	96.18 (13)	O67—Mo7—O57	152.48 (12)
O1B—Mo1—O1M4	154.88 (14)	O7B—Mo7—O2M4	155.59 (14)
O12—Mo1—O1M4	85.24 (11)	O7A—Mo7—O2M4	96.25 (13)
O13—Mo1—O1M4	72.31 (11)	O67—Mo7—O2M4	84.65 (11)
O1A—Mo1—O124	164.91 (13)	O57—Mo7—O2M4	72.44 (11)
O1B—Mo1—O124	87.97 (13)	O7B—Mo7—O467	88.02 (13)
O12—Mo1—O124	73.97 (11)	O7A—Mo7—O467	164.99 (14)
O13—Mo1—O124	83.74 (11)	O67—Mo7—O467	73.65 (11)
O1M4—Mo1—O124	70.99 (10)	O57—Mo7—O467	84.44 (11)
O1A—Mo1—Mo3	88.18 (11)	O2M4—Mo7—O467	71.16 (10)
O1B—Mo1—Mo3	126.50 (11)	O7B—Mo7—Mo5	127.59 (11)
O12—Mo1—Mo3	126.89 (9)	O7A—Mo7—Mo5	88.33 (11)
O13—Mo1—Mo3	34.13 (9)	O67—Mo7—Mo5	126.26 (9)
O1M4—Mo1—Mo3	41.71 (7)	O57—Mo7—Mo5	34.08 (9)
O124—Mo1—Mo3	87.15 (7)	O2M4—Mo7—Mo5	41.66 (7)
O2A—Mo2—O2B	104.26 (15)	O467—Mo7—Mo5	87.32 (7)
O2A—Mo2—O12	99.18 (14)	Mo2—O12—Mo1	114.96 (15)
O2B—Mo2—O12	100.77 (14)	Mo3—O13—Mo1	110.45 (14)
O2A—Mo2—O25	99.57 (14)	Mo3—O1M4—Mo6	97.06 (11)
O2B—Mo2—O25	90.51 (14)	Mo3—O1M4—Mo1	95.78 (11)
O12—Mo2—O25	154.96 (12)	Mo6—O1M4—Mo1	150.49 (15)
O2A—Mo2—O2M4	95.94 (13)	Mo3—O1M4—Mo4	101.69 (12)
O2B—Mo2—O2M4	155.53 (13)	Mo6—O1M4—Mo4	101.69 (11)
O12—Mo2—O2M4	89.29 (11)	Mo1—O1M4—Mo4	101.56 (11)
O25—Mo2—O2M4	72.40 (11)	Mo2—O2M4—Mo5	97.31 (11)
O2A—Mo2—O124	166.43 (13)	Mo2—O2M4—Mo7	151.10 (15)
O2B—Mo2—O124	89.00 (13)	Mo5—O2M4—Mo7	95.61 (11)
O12—Mo2—O124	75.04 (11)	Mo2—O2M4—Mo4	101.50 (11)
O25—Mo2—O124	82.96 (11)	Mo5—O2M4—Mo4	101.59 (11)
O2M4—Mo2—O124	71.98 (10)	Mo7—O2M4—Mo4	101.07 (11)
O2A—Mo2—Mo5	88.60 (11)	Mo5—O25—Mo2	109.26 (14)
O2B—Mo2—Mo5	124.49 (11)	Mo3—O36—Mo6	109.60 (14)
O12—Mo2—Mo5	130.65 (9)	Mo5—O57—Mo7	110.69 (14)
O25—Mo2—Mo5	34.02 (8)	Mo6—O67—Mo7	115.34 (15)
O2M4—Mo2—Mo5	41.36 (7)	Mo4—O124—Mo2	109.78 (13)
O124—Mo2—Mo5	86.20 (7)	Mo4—O124—Mo1	110.17 (12)
O3B—Mo3—O3A	104.96 (16)	Mo2—O124—Mo1	90.79 (10)
O3B—Mo3—O36	98.70 (15)	Mo4—O467—Mo6	109.92 (13)
O3A—Mo3—O36	103.26 (14)	Mo4—O467—Mo7	110.30 (13)
O3B—Mo3—O13	98.61 (14)	Mo6—O467—Mo7	91.11 (10)
O3A—Mo3—O13	98.55 (14)	N31—C1—N21	120.9 (5)
O36—Mo3—O13	147.48 (12)	N31—C1—N11	118.1 (5)
O3B—Mo3—O1M4	104.31 (14)	N21—C1—N11	120.9 (5)
O3A—Mo3—O1M4	150.60 (13)	N32—C2—N12	120.0 (4)
O36—Mo3—O1M4	74.87 (11)	N32—C2—N22	120.1 (4)
O13—Mo3—O1M4	74.28 (11)	N12—C2—N22	119.8 (4)
O3B—Mo3—Mo1	91.43 (11)	N13—C3—N33	119.9 (4)
O3A—Mo3—Mo1	133.64 (11)	N13—C3—N23	120.1 (4)
O36—Mo3—Mo1	116.92 (9)	N33—C3—N23	120.0 (4)
O13—Mo3—Mo1	35.43 (9)	N14—C4—N34	120.1 (4)
O1M4—Mo3—Mo1	42.52 (7)	N14—C4—N24	119.8 (4)
O3B—Mo3—Mo6	92.46 (12)	N34—C4—N24	120.1 (5)
O3A—Mo3—Mo6	138.95 (11)	N15—C5—N35	120.1 (4)
O36—Mo3—Mo6	36.46 (9)	N15—C5—N25	119.3 (4)
O13—Mo3—Mo6	115.49 (9)	N35—C5—N25	120.6 (4)
O1M4—Mo3—Mo6	41.57 (7)	N16—C6—N36	120.1 (4)
Mo1—Mo3—Mo6	81.340 (13)	N16—C6—N26	121.2 (4)
O34—Mo4—O45	104.71 (17)	N36—C6—N26	118.6 (4)
O34—Mo4—O124	101.08 (13)	C1—N11—H11A	120.0
O45—Mo4—O124	100.86 (13)	C1—N11—H11B	120.0
O34—Mo4—O467	101.26 (13)	H11A—N11—H11B	120.0
O45—Mo4—O467	101.58 (13)	C2—N12—H12A	120.0
O124—Mo4—O467	142.93 (14)	C2—N12—H12B	120.0
O34—Mo4—O1M4	83.76 (13)	H12A—N12—H12B	120.0
O45—Mo4—O1M4	171.52 (13)	C3—N13—H13A	120.0
O124—Mo4—O1M4	76.79 (11)	C3—N13—H13B	120.0
O467—Mo4—O1M4	76.67 (11)	H13A—N13—H13B	120.0
O34—Mo4—O2M4	171.58 (12)	C4—N14—H14A	120.0
O45—Mo4—O2M4	83.71 (12)	C4—N14—H14B	120.0
O124—Mo4—O2M4	76.66 (11)	H14A—N14—H14B	120.0
O467—Mo4—O2M4	76.92 (11)	C5—N15—H15A	120.0
O1M4—Mo4—O2M4	87.82 (11)	C5—N15—H15B	120.0
O5B—Mo5—O5A	104.80 (16)	H15A—N15—H15B	120.0
O5B—Mo5—O25	98.17 (14)	C6—N16—H16A	120.0
O5A—Mo5—O25	102.93 (14)	C6—N16—H16B	120.0
O5B—Mo5—O57	98.72 (14)	H16A—N16—H16B	120.0
O5A—Mo5—O57	99.06 (15)	C1—N21—H21A	120.0
O25—Mo5—O57	147.72 (12)	C1—N21—H21B	120.0
O5B—Mo5—O2M4	103.37 (13)	H21A—N21—H21B	120.0
O5A—Mo5—O2M4	151.75 (13)	C2—N22—H22A	120.0
O25—Mo5—O2M4	74.91 (11)	C2—N22—H22B	120.0
O57—Mo5—O2M4	74.52 (11)	H22A—N22—H22B	120.0
O5B—Mo5—Mo7	91.35 (11)	C3—N23—H23A	120.0
O5A—Mo5—Mo7	134.00 (11)	C3—N23—H23B	120.0
O25—Mo5—Mo7	117.25 (9)	H23A—N23—H23B	120.0
O57—Mo5—Mo7	35.23 (9)	C4—N24—H24A	120.0
O2M4—Mo5—Mo7	42.73 (7)	C4—N24—H24B	120.0
O5B—Mo5—Mo2	91.60 (11)	H24A—N24—H24B	120.0
O5A—Mo5—Mo2	139.02 (11)	C5—N25—H25A	120.0
O25—Mo5—Mo2	36.72 (9)	C5—N25—H25B	120.0
O57—Mo5—Mo2	115.50 (9)	H25A—N25—H25B	120.0
O2M4—Mo5—Mo2	41.33 (7)	C6—N26—H26A	120.0
Mo7—Mo5—Mo2	81.460 (12)	C6—N26—H26B	120.0
O6A—Mo6—O6B	105.22 (15)	H26A—N26—H26B	120.0
O6A—Mo6—O67	99.29 (14)	C1—N31—H31A	120.0
O6B—Mo6—O67	100.69 (14)	C1—N31—H31B	120.0
O6A—Mo6—O36	99.78 (15)	H31A—N31—H31B	120.0
O6B—Mo6—O36	91.00 (14)	C2—N32—H32A	120.0
O67—Mo6—O36	154.12 (12)	C2—N32—H32B	120.0
O6A—Mo6—O1M4	95.27 (14)	H32A—N32—H32B	120.0
O6B—Mo6—O1M4	155.62 (14)	C3—N33—H33A	120.0
O67—Mo6—O1M4	88.66 (12)	C3—N33—H33B	120.0
O36—Mo6—O1M4	72.31 (11)	H33A—N33—H33B	120.0
O6A—Mo6—O467	165.28 (13)	C4—N34—H34A	120.0
O6B—Mo6—O467	89.13 (13)	C4—N34—H34B	120.0
O67—Mo6—O467	74.30 (11)	H34A—N34—H34B	120.0
O36—Mo6—O467	82.95 (11)	C5—N35—H35A	120.0
O1M4—Mo6—O467	71.64 (10)	C5—N35—H35B	120.0
O6A—Mo6—Mo3	88.34 (12)	H35A—N35—H35B	120.0
O6B—Mo6—Mo3	124.90 (11)	C6—N36—H36A	120.0
O67—Mo6—Mo3	130.03 (9)	C6—N36—H36B	120.0
O36—Mo6—Mo3	33.93 (8)	H36A—N36—H36B	120.0
O1M4—Mo6—Mo3	41.37 (7)	H1OW—O1W—H2OW	105.7
			
?—?—?—?	?		

### Hydrogen-bond geometry (Å, °)

**Table:**

*D*—H···*A*	*D*—H	H···*A*	*D*···*A*	*D*—H···*A*
N11—H11A···O1W	0.88	1.90	2.765 (6)	168
N11—H11B···O3A	0.88	2.35	3.091 (6)	142
N12—H12A···O2A^i^	0.88	2.00	2.845 (5)	160
N12—H12B···O5B	0.88	2.14	2.903 (5)	145
N13—H13A···O6A^ii^	0.88	1.96	2.828 (5)	169
N13—H13B···O3B	0.88	2.23	2.930 (5)	136
N14—H14B···O13	0.88	1.93	2.774 (4)	159
N14—H14A···O57^iii^	0.88	1.98	2.814 (4)	159
N15—H15A···O2B^iv^	0.88	2.03	2.843 (5)	153
N15—H15B···O3A^v^	0.88	2.13	2.866 (5)	141
N15—H15B···O34^v^	0.88	2.62	3.302 (5)	135
N16—H16B···O1A^ii^	0.88	2.13	2.967 (5)	159
N16—H16A···O45^vi^	0.88	2.18	2.977 (5)	151
N21—H21A···O1B^vi^	0.88	2.18	3.006 (5)	156
N21—H21A···O2B^vi^	0.88	2.40	2.926 (5)	118
N21—H21B···O3A	0.88	2.22	2.992 (5)	147
N22—H22A···O36^vii^	0.88	2.32	3.087 (5)	145
N22—H22A···O467^vii^	0.88	2.50	3.216 (5)	139
N22—H22A···O6B^vii^	0.88	2.64	3.290 (5)	131
N22—H22B···O25	0.88	2.10	2.977 (5)	177
N23—H23A···O36	0.88	2.07	2.937 (5)	169
N23—H23B···O25^vi^	0.88	2.26	3.023 (5)	145
N23—H23B···O124^vi^	0.88	2.50	3.219 (5)	140
N24—H24A···O13	0.88	2.45	3.154 (5)	137
N24—H24A···O3A	0.88	2.50	3.186 (5)	135
N24—H24B···O12^viii^	0.88	2.09	2.855 (5)	145
N25—H25A···O34^v^	0.88	2.22	2.990 (5)	146
N25—H25B···O7A^iii^	0.88	2.08	2.931 (5)	162
N26—H26A···O3B^ii^	0.88	1.98	2.859 (5)	175
N26—H26B···O1W^ii^	0.88	2.50	3.084 (6)	124
N26—H26B···O6A	0.88	2.57	3.197 (5)	129
N31—H31A···O1B^vi^	0.88	2.50	3.248 (6)	143
N31—H31B···O5A^ix^	0.88	2.38	3.188 (6)	152
N32—H32A···N34^x^	0.88	2.50	3.242 (6)	143
N32—H32B···O467^vii^	0.88	2.02	2.864 (5)	160
N33—H33A···N24^vi^	0.88	2.60	3.334 (6)	142
N33—H33B···O124^vi^	0.88	2.02	2.867 (5)	160
N34—H34B···O67^viii^	0.88	1.94	2.776 (5)	157
N34—H34A···O57^iii^	0.88	2.44	3.154 (5)	139
N34—H34A···O5A^iii^	0.88	2.55	3.195 (5)	131
N35—H35A···O2A^iv^	0.88	2.29	3.040 (5)	144
N35—H35B···O5B^iii^	0.88	1.98	2.849 (5)	170
N36—H36A···O5A^vi^	0.88	2.16	2.913 (5)	144
N36—H36B···O6B	0.88	2.13	2.868 (5)	141
O1W—H1OW···O6B^iii^	0.86	2.22	2.948 (5)	142.0
O1W—H1OW···O7B^iii^	0.86	2.44	3.086 (5)	132.8
O1W—H2OW···O5A^ix^	0.86	2.35	2.973 (5)	129.6

Symmetry codes: (i) −*x*+1, −*y*, −*z*; (ii) −*x*, −*y*, −*z*+1; (iii) *x*−1, *y*, *z*; (iv) −*x*, −*y*, −*z*; (v) −*x*, *y*−1/2, −*z*+1/2; (vi) *x*, −*y*+1/2, *z*+1/2; (vii) *x*, −*y*+1/2, *z*−1/2; (viii) −*x*, *y*+1/2, −*z*+1/2; (ix) *x*−1, −*y*+1/2, *z*+1/2; (x) *x*+1, −*y*+1/2, *z*−1/2.
